# The *BDNF* Val66Met polymorphism affects negative memory bias in civilian women with PTSD

**DOI:** 10.1038/s41598-020-60096-1

**Published:** 2020-02-21

**Authors:** Hiroaki Hori, Mariko Itoh, Fuyuko Yoshida, Mingming Lin, Madoka Niwa, Yuko Hakamata, Keiko Ino, Risa Imai, Sei Ogawa, Mie Matsui, Toshiko Kamo, Hiroshi Kunugi, Yoshiharu Kim

**Affiliations:** 1 Department of Behavioral Medicine, National Institute of Mental Health, National Center of Neurology and Psychiatry, Tokyo, Japan; 2 Department of Mental Disorder Research, National Institute of Neuroscience, National Center of Neurology and Psychiatry, Tokyo, Japan; 3 Department of Psychiatry and Cognitive-Behavioral Medicine, Nagoya City University Graduate School of Medical Sciences, Nagoya, Japan; 4 Department of Clinical Cognitive Neuroscience, Institute of Liberal Arts and Science, Kanazawa University, Kanazawa, Japan; 5 Wakamatsu-cho Mental and Skin Clinic, Tokyo, Japan

**Keywords:** Genotype, Genetic predisposition to disease, Post-traumatic stress disorder, Human behaviour

## Abstract

Memory abnormalities are considered a core feature of posttraumatic stress disorder (PTSD). Studies attempting to quantify such memory dysfunction in PTSD have reported that individuals with this disorder exhibit selective memory bias toward negative material. The low expression Met allele of brain-derived neurotrophic factor (*BDNF*) Val66Met polymorphism has been associated with the aetiology of PTSD and with memory abnormalities. It is therefore possible that the *BDNF* Val66Met polymorphism can moderate the relationship between PTSD and memory bias. Here we examined this association in 50 civilian women with PTSD and 70 non-trauma-exposed healthy control women. All subjects were genotyped for the *BDNF* Val66Met (rs6265) polymorphism. Negative memory bias was assessed using a recognition memory task. Patients showed significantly greater negative memory bias compared to controls. In patients, negative memory bias significantly increased with increasing numbers of Met alleles; while no significant relationship was seen in controls. Further pairwise analyses revealed that patients with the Met allele had significantly greater negative memory bias than controls. These results suggest that the relationship between PTSD and negative memory bias can be moderated by the *BDNF* Val66Met polymorphism. More studies are needed to further clarify the relationship between this polymorphism and memory abnormalities in PTSD.

## Introduction

Posttraumatic stress disorder (PTSD) is a serious psychiatric condition that can develop after a major traumatic event, often leading to a chronic course and severe functional impairment. Among various psychobehavioural symptoms of PTSD, involuntary retrieval of traumatic memories that are collectively termed as “re-experiencing” symptoms, such as intrusive thoughts, flashbacks, and nightmares, are postulated as a central feature of this disorder. The presence of these re-experiencing phenomena suggests that trauma-related fear can be easily activated in these individuals even in the absence of an actual threat, and this sense of threat is considered to arise, at least in part, as a consequence of excessively negative appraisals of the trauma and its sequelae^1^.

One approach to quantifying such disordered memory/cognition in PTSD is to experimentally measure selective memory bias toward negative and/or emotionally threatening material. To date, a number of studies have observed the memory bias in individuals with this disorder compared to healthy controls^2–4^. We have also found that female patients with PTSD as a group display negative memory bias, albeit with considerable inter-individual variation^5^. Such variation in negative memory bias may be related to the heterogeneous nature of this disorder.

Evidence suggests that genetic factors are involved in the aetiology of PTSD^6^. Based on both biological mechanisms and data-driven approaches, the brain-derived neurotrophic factor (*BDNF*) gene is considered as one of the most relevant candidate genes for this disorder^6–11^. *BDNF* encodes a neurotrophin that plays a role in neuronal growth, differentiation, maturation and survival in immature neurons and in synaptic plasticity, neurotransmission, and regulation of receptor sensitivity in mature neurons^12^. BDNF and its high affinity receptor tropomyosin-related kinase B (TrkB) are highly expressed in brain regions associated with memory functions, including the hippocampus, amygdala, and prefrontal cortex^7^. A functional single nucleotide polymorphism (SNP) of *BDNF*, Val66Met (rs6265), results in an amino acid substitution from valine (Val) to methionine (Met) at codon 66. The Met substitution is shown to impair intracellular trafficking, reduce activity-dependent secretion of BDNF, and negatively affect human memory and hippocampal function^13^. Using a mouse model with the humanized *BDNF* Val66Met polymorphism knocked-in, it was shown that chronic activation of glucocorticoid receptors during late adolescence increases fear memory in adulthood according to the Val66Met genotype^14^. This finding suggests that chronic stress potentiates the fear circuitry into adulthood in the Met carriers, thereby increasing their susceptibility to fear- and anxiety-related psychiatric disorders such as PTSD^8,14^. With this transgenic mouse model, the same research group showed that the Val66Met polymorphism acts as a regulator of glucocorticoid-induced changes in stress sensitive molecules across corticohippocampal regions^15^. In line with this, a study investigated extinction of conditioned fear responses in genetic knock-in mice as well as humans in order to understand the effect of this polymorphism, demonstrating that the extinction learning was impaired in Met allele carriers relative to non-Met allele carriers for both mice and humans^16^. Another translational study reported that BDNF levels are altered not only in the plasma of PTSD patients carrying the Met allele but also in the plasma and hippocampus of acutely stressed rats^17^. Furthermore, a human study demonstrated that PTSD patients with the Met allele exhibit impaired fear extinction learning compared to those with the Val/Val genotype, suggesting that this polymorphism can cause memory abnormalities in this disorder^18^. However, no studies to date have examined its relationship with memory bias in PTSD.

This study aimed to examine the association of the *BDNF* Val66Met polymorphism with memory bias in civilian women with PTSD and healthy control women. The main reason for the focus on female patients was that this study built on our previous study of memory bias in PTSD women^5^. In addition, it was necessary to consider potential sex differences in the present study, given the evidence for sexually dimorphic effects of the *BDNF* Val66Met polymorphism^12^ and for a role of oestrogen in the expression of *BDNF*^19^. Our primary hypothesis was that this polymorphism would moderate the relationship between PTSD and negative memory bias in a manner that the Met allele would be associated with increased memory bias in patients. Additionally, we examined an association of the *BDNF* Val66Met polymorphism with memory performance in order to more broadly understand the association between this polymorphism and memory abnormalities in PTSD.

## Results

### Sample characteristics

A total of 50 civilian women with PTSD and 70 non-trauma-exposed healthy control women participated in this study. Demographic characteristics, memory bias scores, and cognitive performance in patients and controls are summarized in Table 1. Mean age for patients and that for controls were both mid-to-late thirties, although there was a significant difference. Compared to controls, patients demonstrated significantly greater negative memory bias and poorer memory/cognitive performance. Clinical characteristics of the patients are presented in Table 2. Most patients developed PTSD after experiencing interpersonal violence such as physical and/or sexual violence, and were chronically ill. Many of the patients had psychiatric comorbidity, and were receiving psychotropic medications. The patients were moderately severely ill as indexed by the mean total score of a symptom assessment scale.

**Table 1:** Demographic characteristics, memory bias scores, and cognitive performance in PTSD patients and healthy controls.

	PTSD patients (n = 50)	Healthy controls (n = 70)	p (patients vs. controls)
Age, years: mean ± standard deviation	39.6 ± 8.8	34.4 ± 13.2	**0.011**
Education level^a^: median (25–75th percentile)	3 (2.8–4.0)	3 (3.0–4.0)	0.14
Smoking: yes, n (%)	8 (16.0)	4 (5.7)	0.06
Recognition memory task: median (25–75th percentile)			
Hits, Negative	0.890 (0.780–1.000)	0.890 (0.780–1.000)	0.34
Hits, Neutral	0.780 (0.670–0.890)	0.890 (0.670–1.000)	**0.018**
Negative memory bias	0.111 (0.000–0.333)	0.000 (−0.028–0.111)	**<0.001**
RBANS: mean ± standard deviation			
Immediate memory^b^	83.9 ± 20.5	98.5 ± 13.7	**<0.001**
Visuospatial construction	94.9 ± 14.4	100.6 ± 10.2	**0.018**
Language	98.4 ± 20.1	109.5 ± 14.1	**0.001**
Attention	93.8 ± 17.6	106.3 ± 14.6	**<0.001**
Delayed memory^b^	91.4 ± 18.4	101.9 ± 13.4	**<0.001**
Total score^b^	87.3 ± 23.0	105.4 ± 13.5	**<0.001**

Abbreviations: PTSD, posttraumatic stress disorder; RBANS, Repeatable Battery for the Assessment of Neuropsychological Status.

Notes: Bold p values represent significant results.

T-test was used to compare age and RBANS scores between groups; Mann-Whitney U test was used to compare education level and recognition memory task scores; and χ^2^ test was used to compare smoking status.

^a^Coded as follows: 1, junior high school graduate; 2, high school graduate; 3, some college graduate/partial university; 4, university graduate; 5, graduate school graduate.

^b^n = 69 for controls.

**Table 2:** Clinical variables for patients with PTSD.

Variable	PTSD patients (n = 50)
Outpatients/inpatients: n/n	49/1
Duration of illness^a^: n/n/n/n	3/7/6/34
Type of index trauma: yes, n (%)	
Interpersonal violence	43 (86.0)
Accident	3 (6.0)
Other	4 (8.0)
Comorbid psychiatric disorder, any: yes, n (%)	36 (72.0)
Major depressive disorder	30 (60.0)
Bipolar disorder	3 (6.0)
Anxiety disorder	23 (46.0)
Obsessive-compulsive disorder	6 (12.0)
Alcohol/substance abuse or dependence	7 (14.0)
Medication, any: yes, n (%)	39 (78.0)
Antipsychotics	14 (28.0)
Antidepressants	30 (60.0)
Anxiolytics	28 (56.0)
Mood stabilizers	6 (12.0)
Hypnotics	20 (40.0)
PDS, total score: mean ± standard deviation	32.0 ± 9.5
Intrusion	8.3 ± 3.3
Avoidance	14.0 ± 4.5
Hyperarousal	9.6 ± 3.4

Abbreviations: PTSD, posttraumatic stress disorder; PDS, posttraumatic diagnostic scale.

Notes: ^a^Categorized as follows: “less than 6 months”/“6 months to 3 years”/“3 to 5 years”/“5 years or more”. One patient chose two options (“3 to 5 years” and “5 years or more”), and we coded it as “5 years or more”.

To understand the relationships among different indices pertaining to memory function, we calculated correlations of Hits for negative words (i.e., “Hits, negative”) and those for neutral words (i.e., “Hits, neutral”) with immediate memory and delayed memory scores, separately for patients and controls. This analysis revealed that “Hits, negative” was not significantly correlated with immediate memory or delayed memory in either patients or controls (all p > 0.05) while “Hits, neutral” was significantly positively correlated with immediate memory and delayed memory in both patients or controls (all p < 0.05).

Genotype frequencies did not deviate from Hardy-Weinberg equilibrium in controls [χ^2^(1) = 0.51, p = 0.48] or in patients [χ^2^(1) = 0.19, p = 0.66]. Numbers of Val/Val, Val/Met, and Met/Met genotypes were 17 (34.0%), 23 (46.0%), and 10 (20.0%) for patients and 25 (35.7%), 36 (51.4%), and 9 (12.9%) for controls, respectively.

### *BDNF* Val66Met polymorphism and memory bias

**Figure 1:**
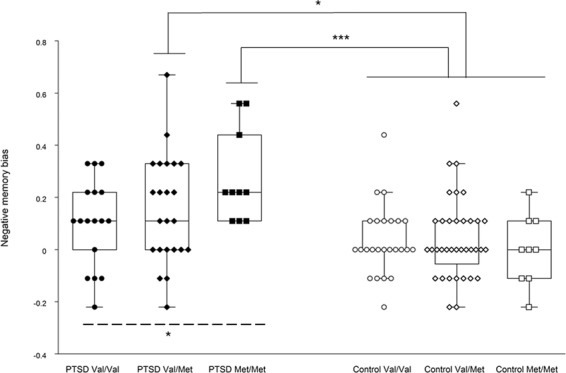
Negative memory bias stratified by the *BDNF* Val66Met genotype groups in patients and controls. Combined dot- and box-plot shows the negative memory bias scores (as calculated by subtracting the hit rates for neutral items from those for negative items) for PTSD patients with the Val/Val (n = 17), Val/Met (n = 23), and Met/Met (n = 10) genotypes and for healthy controls with the Val/Val (n = 25), Val/Met (n = 36), and Met/Met (n = 9) genotypes. *p < 0.05, ***p < 0.001; Val/Val patients, Val/Met patients, Met/Met patients, and total healthy controls were compared using the Kruskal-Wallis test with post-hoc pairwise comparisons with Bonferroni correction. The broken line with an asterisk (*) indicates a significant trend towards greater negative memory bias with increasing numbers of Met alleles in patients, as revealed by the Jonckheere-Terpstra trend test.

Figure 1 shows negative memory bias scores stratified by the *BDNF* Val66Met genotype groups in patients and controls. In patients, the Jonckheere-Terpstra trend test showed that negative memory bias significantly increased with increasing numbers of Met alleles (JT = 503.0, p = 0.049); while no significant relationship was seen in controls (JT = 694.0, p = 0.72). The Kruskal-Wallis test with Bonferroni-corrected post-hoc pairwise comparisons revealed that patients with the Val/Met genotype (p = 0.045) and those with the Met/Met genotype (p < 0.001), but not those with the Val/Val genotype (p = 0.53), had significantly greater negative memory bias compared to total controls (in this analysis, control subjects were grouped together since no allele effect was seen in this group).

### *BDNF* Val66Met polymorphism and memory performance

**Figure 2:**
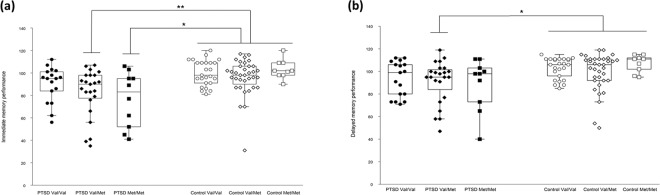
Immediate (**a**) and delayed (**b**) memory performance stratified by the *BDNF* Val66Met genotype groups in patients and controls. Combined dot- and box-plot shows the (**a**) immediate memory index scores and (**b**) delayed memory index scores (as measured by the Repeatable Battery for the Assessment of Neuropsychological Status) for PTSD patients with the Val/Val (n = 17), Val/Met (n = 23), and Met/Met (n = 10) genotypes and for healthy controls with the Val/Val (n = 25), Val/Met (n = 35), and Met/Met (n = 9) genotypes. *p < 0.05, **p < 0.01; Val/Val patients, Val/Met patients, Met/Met patients, and total healthy controls were compared using the Kruskal-Wallis test with post-hoc pairwise comparisons with Bonferroni correction.

Figure 2 shows immediate memory and delayed memory performance stratified by the *BDNF* Val66Met genotype groups in patients and controls. For immediate memory, the Jonckheere-Terpstra test showed no significant trend in patients (JT = 313.0, p = 0.13) or in controls (JT = 725.5, p = 0.84). However, the Kruskal-Wallis test with Bonferroni-corrected post-hoc pairwise comparisons revealed that Val/Met patients (p = 0.002) and Met/Met patients (p = 0.02), but not Val/Val patients (p = 0.31), had significantly poorer immediate memory performance than total controls. For delayed memory, the Jonckheere-Terpstra test showed no significant trend in patients (JT = 370.5, p = 0.65) or in controls (JT = 765.5, p = 0.50). The Kruskal-Wallis test with Bonferroni-corrected post-hoc pairwise comparisons revealed that Val/Met patients (p = 0.026), but not Val/Val patients (p = 0.20) or Met/Met patients (p = 0.30), had significantly poorer delayed memory performance than total controls.

### Symptomatology and potentially confounding variables in patients

In patients, PTSD severity, including intrusion (Kruskal-Wallis H = 2.6, p = 0.28), avoidance (H = 5.1, p = 0.08), hyperarousal (H = 3.5, p = 0.18), and the total score (H = 5.7, p = 0.06), was not significantly different among the three genotype groups.

Age was significantly different between patients and controls. However, age was not significantly different among the three genotype groups in patients [F(2,47) = 0.29, p = 0.75]; mean age of patients with the Val/Val, Val/Met, and Met/Met genotypes was 40.9 ± 7.4, 38.7 ± 8.5, and 39.6 ± 12.1 years (respectively). Age did not significantly differ among the four groups, i.e., three genotype groups in patients plus one control group, either [F(3,116) = 2.05, p = 0.11]. In addition, age was not significantly correlated with negative memory bias in patients (rho = −0.047, p = 0.75).

To examine the effects of other potential confounders including education, smoking, menstrual cycle, psychiatric comorbidities, and medication, we tested whether these variables were significantly different among the three genotype groups in patients. These three groups did not significantly differ in education levels (Kruskal-Wallis H = 0.27, p = 0.87) or smoking status (Fisher’s exact test: p = 0.33). For menstrual cycles, the numbers of patients who endorsed “before menstruation (within 1 week)”, “during menstruation”, “after menstruation (within 1 week)”, and “after menopause” did not significantly differ among the three groups (Fisher’s exact test: p = 0.38). The three groups did not significantly differ in the presence/absence of comorbid major depressive disorder (Fisher’s exact test: p = 0.14) or anxiety disorder (Fisher’s exact test: p = 0.46). The groups did not significantly differ in the use of antipsychotics (Fisher’s exact test: p = 0.19), antidepressants (Fisher’s exact test: p = 1.0), anxiolytics (Fisher’s exact test: p = 0.24), or hypnotics (Fisher’s exact test: p = 0.12).

## Discussion

This study, which extends our previous finding of memory bias^5^, is the first, to our knowledge, to investigate the association between the *BDNF* Val66Met polymorphism and memory bias in PTSD. As hypothesized, we observed a significant relationship of Met alleles with greater negative memory bias in patients. Furthermore, our results showed that, among patients, only Met allele carriers but not Val/Val homozygotes have greater negative memory bias than healthy controls.

Genetic studies for PTSD are useful to identify risk factors for the development of the disorder and improve its treatment^20^, and *BDNF* is considered a relevant candidate gene for PTSD^6–8,21^. Supporting this, previous studies have shown that Met allele carriers of the *BDNF* Val66Met polymorphism, compared to Val/Val homozygotes, have impaired fear extinction learning^18^ and poorer response to exposure therapy^22^. Our findings also accord with those of animal model studies for PTSD that demonstrated the role of the Val66Met polymorphism in the fear memory circuitry^14^. The present finding thus adds to the literature on the effect of this polymorphism on memory abnormalities associated with PTSD. In line with our finding, it was reported that among healthy adults with childhood stressful events, Met allele carriers tended to recall a lower proportion of positive words compared to Val/Val homozygotes^23^.

Our results also suggested that the Met allele of this polymorphism could be associated with lower memory performance in PTSD since the pairwise analysis revealed significant differences against controls, although the trend-test result was not significant. Such impairment in memory performance associated with Met allele of the *BDNF* Val66Met polymorphism accords with previous findings^13^. It should be noted, however, that the overall poorer cognitive performance as revealed by total RBANS scores in the PTSD patients, on top of the effect of the *BDNF* genotype, could have contributed to the result of significantly worse memory performance in the Met allele-carrying patients than in controls. Furthermore, the association of the Val66Met with memory performance in our patients was less clear than that with memory bias, suggesting that, in PTSD, this polymorphism could be involved more in the core memory abnormality of this disorder than in general memory dysfunction. This possibility is supported by the findings of several previous studies, including those in rodents^10,14^, in humans^11,18^, and in both^16,17^.

While most previous studies conducted among Caucasians have combined *BDNF* Met/Met homozygotes with Val/Met heterozygotes in their analyses^11,18,23–25^, they were separately analysed in the present study for Japanese subjects. This difference can be attributable to the fact that the minor Met allele frequency was relatively higher in this study (or in the Japanese population in general) than in the previous studies among Caucasian. Specifically, the Met allele frequency in our subjects was 0.43 for PTSD patients and 0.39 in healthy controls, and in line with this, it is reported to be 0.41 in a representative genome variation database of Japanese individuals^26^. In contrast, the Met allele frequency is reported to be no more than 0.18 in European populations and 0.15 in American populations, according to the Genome Aggregation Database (gnomAD). Meanwhile, it is pointed out that the genotype grouping of Val/Met and Met/Met into a single group may not be an ideal way to examine the effect of this polymorphism because 1) there is an ethnicity bias within the literature where all three genotype groups have been assessed, 2) genotype effects, relative to allelotype effects, have been understudied, and 3) a dosage effect of Met allele has been demonstrated in animal studies^8^.

There were several limitations to the current study. First, the sample size was relatively small, and therefore some of the negative results may have actually represented type II errors. For example, the absence of a significant association between the *BDNF* genotype groups and memory bias/performance in controls might be attributable to the type II error owing to small sample size; however, it was also possible that this result may have been negative because the control subjects collectively had less bias and higher performance than patients. Another possibility would be related to the differential mean age between patients and controls, that is, patients were approximately five years older than controls. Given the evidence that aging can magnify genetic effects on cognitive function^27^, the significant effect of the *BDNF* Val66Met polymorphism on memory function observed only in patients but not in controls may be accounted for by the relatively advanced age of patients. Furthermore, various patient-specific variables such as comorbid psychiatric disorders and psychotropic medications might have also exaggerated the effect of *BDNF* Val66Met on memory bias and function in the PTSD group. Second, it was difficult to determine whether the memory abnormalities in patients were associated with the PTSD illness or with the trauma exposure itself, as the present sample did not include trauma-exposed healthy individuals without PTSD. Third, the present study included only female participants, which may have affected the findings. For instance, it has been reported that the *BDNF* Val66Met polymorphism can have sexually dimorphic effects on the susceptibility to depression^28^ and Alzheimer’s disease^29^. Oestrogen is shown to induce the expression of *BDNF* through its receptors that co-localize with BDNF-synthesizing neurons^19^. Ovarian hormones can also be involved in intrusive memories and fear extinction^30,31^. In addition, the prevalence of PTSD has been consistently shown to be higher in women than in men^32^. The heritability of PTSD can also differ between men and women, such that this heritability is generally estimated at around 30–40%^33^ whereas it can be as high as 72% when the sample was restricted to female patients^34^. Taken together, our finding of an association between the *BDNF* Val66Met polymorphism and memory bias might represent certain unique feature of women with PTSD. Without male subjects, however, the present study does not allow any conclusions to be drawn about this association in PTSD men. Finally, as Japanese kanji characters were used in the recognition memory task, our findings may not be fully generalizable to other ethnicities. Still, however, the significantly greater negative memory bias in PTSD patients than in controls was consistent with the findings from previous studies^2–4^, which suggests that the memory bias in PTSD can be constantly observed across different tasks and languages. In addition, the words used in this task were chosen from a corpus of Japanese two-character kanji compounds^35^, in which affective valence rating scores for these words were determined^35^.

In conclusion, this study shows that the relationship between PTSD and memory bias is moderated by the *BDNF* Val66Met polymorphism such that the Met allele leads to an increased bias. These results provide support to the accumulated evidence on the role of *BDNF* gene in psychiatric disorders associated with memory abnormalities. More studies are needed to further clarify the relationship between this polymorphism and memory abnormalities in PTSD.

## Methods

### Participants

Details of participant recruitment have been reported previously^5^. Briefly, 50 civilian women with PTSD (age range: 23–56 years) and 70 non-trauma-exposed healthy control women (20–64 years) participated in this study. This sole inclusion of females (or exclusion of males) in the previous and present studies was because the vast majority of the sample included in our entire project were females, with only four male subjects (two patients and two controls) having been enrolled as of November 2019; this was due to the fact that the potentially eligible PTSD patients who were visiting the hospitals/clinics affiliated with the research institutes for this study were mostly females.

All participants were native Japanese speakers who resided in metropolitan areas in Japan and had no severe physical illness or apparent intellectual disability. All patients had already been diagnosed as having PTSD by their attending clinicians. The experience of traumatic events and diagnosis of PTSD were confirmed by the validated Japanese version^36^ of the Posttraumatic Diagnostic Scale (PDS)^37^. The PDS also assesses PTSD severity during the past month reflecting Criteria B-D, i.e., intrusion, avoidance, and hyperarousal, as well as the total score. For healthy controls, the PDS was administered to examine the presence/absence of traumatic experiences and, if present, they were excluded from this study. In addition, the Mini International Neuropsychiatric Interview (M.I.N.I.)^38^ was administered to identify any other Axis-I disorders as well as PTSD in patients and to ascertain the absence of any Axis-I disorders in controls.

This study was approved by the ethics committees of three institutes involved, namely National Center of Neurology and Psychiatry ethics committee, Tokyo Women’s Medical University ethics committee, and Nagoya City University ethics committee, and was conducted in accordance with the Declaration of Helsinki. Written informed consent was obtained from all participants after they had received a detailed explanation of the study. A significant subset of the present participants (95 of the total 120 participants: 79.2%) had been included in our previous study on the association between memory bias and memory performance^5^.

### Measurement of negative memory bias

The recognition memory task was used to assess memory bias, as described previously^5^. Briefly, this task comprised an encoding phase and a delayed-recognition test phase. Twenty-seven words were used in the encoding phase, with nine items each for negative, neutral, and positive words. In the delayed-recognition test phase, a total of 54 words were presented; half of them were the “old” items and the remaining half were “new” ones.

In this study, we focused on the primary outcome index of this task, i.e., “negative bias for hits” as calculated by subtracting the hit rates for neutral items (i.e., “Hits, neutral”) from those for negative items (i.e., “Hits, negative”); for this index, a significant difference between PTSD patients and controls had been observed in our previous study^5^.

On the other hand, these two indices of the recognition memory task, i.e., “Hits, neutral” and “Hits, negative”, were not used as a measure of memory performance. The reason for this was that these indices were not considered as suitable as the Repeatable Battery for the Assessment of Neuropsychological Status (RBANS)^39,40^ for representing memory performance because the former, unlike the latter, was created to measure emotionally valenced (vs. unvalenced) memory, and moreover, not standardized.

### Measurement of memory performance

The RBANS^39,40^, a well-established neuropsychological test battery, was used to assess cognitive functions including memory performance. With 12 subtests, the RBANS can assess immediate memory, visuospatial construction, language, attention, and delayed memory, as well as the total score. Age-corrected standardized scores, with a population mean of 100 and standard deviation (SD) of 15, are calculated for each cognitive domain^39,40^. It was carried out on the same day as the recognition memory task.

In the present analysis, we focused on the two memory indices of RBANS, namely immediate memory and delayed memory. Of the total 120 subjects, one healthy control subject failed to complete this test battery.

### Genotyping

Genomic DNA was prepared from venous blood according to standard procedures. Rs6265 (Val66Met) was genotyped using the TaqMan SNP Genotyping Assays (assay ID: C__11592758_10). The thermal cycling conditions for polymerase chain reaction were: 1 cycle at 95 °C for 10  min followed by 45 cycles of 95 °C for 15 s and 60 °C for 1  min. The allele-specific fluorescence was measured with ABI PRISM 7900 Sequence Detection Systems (Applied Biosystems, Foster City, CA). All samples had a genotyping call rate of 97% or greater.

### Statistical analysis

Averages are reported as “means ± SD”, or “median (25–75th percentile)” where appropriate. The t-test or Mann-Whitney U test was used to examine differences between two groups. The analysis of variance or Kruskal-Wallis test was used to examine differences among three groups. The use of parametric or nonparametric test was determined based on the nature and distribution of data. Categorical variables were compared using the χ^2^ test, or Fisher’s exact test where appropriate. Correlations of the recognition memory task indices with other variables were calculated using Spearman’s rank order correlation (rho) since the former data did not follow the normal distribution.

Relationships of the *BDNF* Val66Met polymorphism with memory bias/function were examined using nonparametric tests, given that memory bias scores did not follow the normal distribution and that sample sizes for each genotype subgroup were small. Specifically, the Jonckheere-Terpstra trend test was performed to examine the relationship between the three genotypes (i.e., Val/Val vs. Val/Met vs. Met/Met) and memory bias/function within each diagnostic group. We used this trend test as a screening analysis, considering the possibility that memory abnormalities would become greater with an increasing number of Met alleles. The Kruskal-Wallis test, along with post-hoc pairwise analyses controlling for multiple tests, was also performed to make direct comparisons between groups.

In addition, we compared symptom severity (as assessed by the PDS) between the three genotype groups within patients, using the Kruskal-Wallis test. We also examined the effects of potentially confounding variables, including demographic and clinical characteristics, on the main outcomes. Specifically, age was compared among the three genotype groups in patients and among the four groups (i.e., three genotype groups in patients plus one control group), using the analysis of variance. Additionally, education levels, smoking status, menstrual cycles, comorbid psychiatric disorders, and psychotropic medications were compared among the three genotype groups in patients.

Statistical significance was set at two-tailed p < 0.05. All statistical analyses were performed using the Statistical Package for the Social Sciences version 25 (IBM Corp., Tokyo, Japan).

## Data Availability

The data that support the findings of this study are available from the corresponding author, H.H., upon reasonable request.
